# The Roles of Anandamide, Fatty Acid Amide Hydrolase, and Leukemia Inhibitory Factor on the Endometrium during the Implantation Window

**DOI:** 10.3389/fendo.2017.00268

**Published:** 2017-10-16

**Authors:** Na Cui, Changyan Wang, Zhiming Zhao, Jie Zhang, Yueming Xu, Yang Yang, Guimin Hao

**Affiliations:** ^1^Department of Reproduction, The Second Hospital of Hebei Medical University, Shijiazhuang, China; ^2^Department of Reproduction, Handan Center Hospital of Hebei Province, Handan, China

**Keywords:** anandamide, endocannabinoid system, endometrial receptivity, fatty acid amide hydrolase, unexplained infertility, leukemia inhibitory factor

## Abstract

**Background/aims:**

We investigated the role of the endocannabinoid system (ECS) in the endometrium of unexplained infertility (UI) patients, and effect of anandamide (AEA) on leukemia inhibitory factor (LIF).

**Methods:**

Patients were divided into UI and control groups. Endometrium samples were collected at the midluteal phase. Levels of cannabinoid type 1 (CB1), fatty acid amide hydrolase (FAAH), and LIF were examined. LIF productions were measured after AEA, CB1 antagonist AM251, and CB2 antagonist AM630 stimulation.

**Results:**

Rates of available embryo, successful implantation and pregnancy, and the endometrial thickness of UI group were significantly lower than control, suggesting uterine receptivity was decreased in UI group. FAAH and LIF levels were significantly decreased, whereas endometrial CB1 was slightly increased in UI group. LIF production was promoted by low amount of AEA administration (1–10 μM), while the promotion was reduced by higher concentration of AEA (50 μM). LIF levels were decreased by AM251 or AM630, compared with AEA alone. Expressions of FAAH and LIF were closely associated with uterus receptivity and implantation rate of UI patients. Different concentrations of AEA could stimulate dynamic changes in LIF production.

**Conclusion:**

Our data indicated the important role of the ECS in human fertility, which may promote new strategies for successful implantation and treatments for reproductive diseases.

## Introduction

The unexplained infertility (UI) was proposed in 1960s, but efficient treatments are yet to be developed today (1). Many couples still have no explanation for their infertility even after advanced diagnostic assessment, and up to 10–20% of infertile couples have UI (2). The possible reasons could be that the ovum is not released at the optimum time for fertilization, the sperm is difficult to access to the ovum, or the embryo fails to implant into the endometrium (3). It is increasingly recognized that the embryonic implantation is of critical importance for successful fertilization (4). The uterus exhibits maximal receptivity for random lying blastocyst in the implantation window phase. This period of receptivity is transient and results from the programmed sequence of the effect of sex hormones and cytokines on the endometrium. The disturbance of intrauterine environment might lead to the decreased uterine receptivity, which is one of the causes of the UI.

The endocannabinoid system (ECS) has been implicated in the uterine receptivity, blastocyst implantation, maintenance of early pregnancy, and embryo development (5). The ECS is composed of the endocannabinoids, cannabinoid receptors, and a variety of related enzymes. Two important endogenous cannabinoid ligands, *N*-arachidonoyl-ethanolamine (anandamide, AEA) and 2-arachidonoyl-glycerol (2-AG), have been identified in 1990s (6, 7), and a number of additional endocannabinoids were found several years later, including 2-arachidonyl-glyceryl ether (2-AGE), *O*-arachidonoyl-ethanolamine (virodhamine), and *N*-arachidonoyl-dopamine (NADA) (8). AEA is present in the uterus, affecting the receptivity between the blastocyst and uterine during the implantation window, and the development of preimplantation embryo, which is important for a successful implantation (9). Several studies revealed that the AEA levels were lower in receptive uteri but higher in nonreceptive uteri (10). The endocannabinoids exert their actions through their G protein-coupled receptors: cannabinoid receptor type 1 (CB1) and CB2. The activation of CB1 by AEA promoted the adsorption of sperm by the fallopian tube, resulting in the sperm migration to the fertilization (11). In addition, CB1-deficient females were subfertile with oviductal retention of blastocysts (12). The levels of endocannabinoids are tightly regulated, and recent years have seen the identification of a number of enzymes involved in their synthesis and degradation. AEA is degraded by the enzyme fatty acid amide hydrolase (FAAH) or the lysosomal *N*-acylethanolamine-hydrolyzing acid amidase, which metabolizes it into amino acids and ethanolamine (13). It was reported that the FAAH activity is crucial for uterus–embryo crosstalk, and deletion of FAAH led to persistently high AEA levels, resulting in defective implantation (13). In addition to having a role in the uterus, the FAAH present in peripheral T cells has a crucial role in controlling pregnancy (14). Normal gestation is dependent on a delicate controlled adaptive immune response, which involves the release of type 2 T-helper (Th2) cytokines (IL-3, IL-4, and IL-10) (15) and leukemia inhibitory factor (LIF) (16). Th2 cytokines promote trophoblast growth by inhibition of natural killer cells activity. LIF is a member of IL-6 family that promotes embryo implantation and survival (17). The decreased expression and activity FAAH in T cells led to the increased level of AEA in blood, and the excessive AEA dramatically reduces the production of LIF by T cells *via* a CB1 receptor-dependent mechanism, resulted in spontaneous abortion in the early stages (18, 19). LIF gene mutation in infertile women was significantly increased and had a negative impact on the *in vitro* fertilization (IVF) outcome (20). Recent studies evaluated LIF expression in patients with UI, tubal factor, poor ovarian reserve, and endometriosis. They found LIF expression was significantly impaired in UI group compared with the control group, while no significant difference was observed in other infertility sub-groups (21). These studies demonstrated that the ECS and LIF are essential for the implantation and maintenance of the fetus. However, the effects of ECS on the intrauterine environment during the implantation window, the cross-talking between ECS and LIF, and the mechanisms of ECS and LIF on human unexplained fertility were not completely understood.

In this study, we studied the expressions of FAAH, CB1, and LIF in the endometrial tissue of UI patients during the implantation window. Further analysis investigated whether AEA have a regulating function on LIF production. Our research into ECS function helped us to better understand molecular signaling networks involved in embryo implantation and to develop new strategies to improve pregnancy outcome conceived from IVF.

## Materials and Methods

### Patients and Grouping Strategy

A blinded study was performed on the patients who proposed to perform IVF treatment in the assisted reproductive center, Second Hospital of Hebei Medical University from March 2014 to December 2014. A total of 28 patients was selected to form the UI group with the following criteria: under the age of 38, menstruating in regular cycles (25–35 days), having spontaneously ovulation and passable bilateral tubal, having no pregnancy after receiving at least three times of intrauterine insemination (IUI), excluding the possible of uterine, endocrine or immune disturbance and the male factor, without endometriosis symptom, not taking any medication, or hormonal contraception for at least 3 months before recruitment into the studies.

The control group included 32 patients who proposed to perform IVF/intracytoplasmic sperm injection (ICSI) treatment in our center at the same period, due to the male factor or tubal occlusion (except for the hydrosalpinx). The following criteria were used: under the age of 38, having previous pregnancies, menstruating in regular cycles (25–35 days), having spontaneously ovulation and normal basic hormones levels, not taking any medication or hormonal contraception for at least 3 months before recruitment into the studies, excluding the possible of polycystic ovary syndrome, endometriosis, hysteromyoma, uterine endometrial polyps, and other diseases. The individual in this manuscript has given written informed consent to publish these case details. This study was approved by the ethics committee of Handan Center Hospital of Hebei Province. All procedures performed in studies involving human participants were in accordance with the ethical standards of the institutional and/or national research committee and with the 1964 Helsinki Declaration and its later amendments or comparable ethical standards.

### Specimen Collection

To evaluate the ovulation day, both of the two groups were received ovulation detection *via* transvaginal b-ultrasonography (Prosound α7, Aloka, Japan) 1 week before IVF/ICSI treatment. On the day 7–9 after ovulation, a blood sample (5 mL) was taken from each patient. The blood was left to clot for 15 min before being centrifuged at 1,500 *g* for 10 min, and then separated serum was stored at −80°C for later measurements. In addition, on the day 7–9 after ovulation, we detected the endometrial thickness by transvaginal b-ultrasonography, and then scraped the endometrium tissues of the patients. The fresh endometrium tissues were fixed with 4% paraformaldehyde for 24 h, followed by dehydration, paraffin embedding, and serial section (4 µm) before test.

The patients’ physical condition were adjusted by the injection (subcutaneously) of gonadotropin (Gn)-releasing hormone agonist (GnRH-a, 0.1 mg/day) for 18 days, starting on the day of having curettage surgery. After the pituitary downregulation by GnRH-a, the patients were administrated with Gn when the sex hormone level dropped to the designed standard value. The patients were treated with human chorionic gonadotropin (HCG, 10,000 IU) when three or more follicles (diameter ≥18 mm) appeared. After 36 h, the eggs were taken by transvaginal ultrasound-guided puncture, followed by IVF/ICSI according to the sperm treating process or previous fertilization conditions. The patients were given progesterone (40 mg) injection intramuscularly on the day of picking up eggs, and then they were daily injected with progesterone (60 mg/day). After 14 days of transplantation, the patients were examined of β-HCG. The β-HCG-positive patients were given transvaginal ultrasound examination in 30 days after transplantation. The observation of gestational sac was the indication of clinical pregnancy, including intrauterine and extrauterine pregnancy.

### Cell Culture

The human endometrial adenocarcinoma cell line RL95-2 was obtained from the Chinese Academy of Sciences Committee Type Culture Collection cell bank. The cells were grown in Dulbecco’s modified Eagle’s medium/F12 (Gibco, Grand Island, NY, USA) supplemented with 10% fetal bovine serum (Biological, Beit Haemek, Israel) and 1% penicillin and streptomycin (Bioind, Beit Haemek, Israel) at 37°C. The culture medium was changed every second day, and let the cells grow up to 80% confluency before passaging. The cells were washed by PBS (Gibco) once, followed by incubation with 0.25% Tyrisin–EDTA (Invitrogen, Carlsbad, CA, USA) at 37°C for 3 min and collection.

### Cytokine Production Assays

The RL95-2 cells (5 × 10^6^ cells/mL) were seeded in the flasks and stimulated with AEA (5, 10, and 50 µmol/L, Sigma, St. Louis, MO, USA), AM251 (5 µmol/L, Sigma), or AM630 (5 µmol/L, Tocris, UK) alone, and AEA together with AM251 or AM630 for various periods of time. The supernatants were collected at 24, 48, and 72 h, followed by centrifugation at 800 *g* for 8 min, and then LIF were measured by ELISA kit (Bio-swamp, Shanghai, China).

### HE Staining

Paraffin sections (4 mm) were cut from endometrial tissue, deparaffinized in xylene, and rehydrated in graded alcohol. The detailed process was as follows: the sections were soaked in xylene I and xylene II for 5 min each, and subsequently soaked in 100, 95, 80, and 75% ethanol for 1 min each, and then washed by distilled water for 2 min. The specimens were stained with hematoxylin for 5 min, and eosin for 2 min, followed by water flushing. The slices were then dehydrated by 95 and 100% ethanol, hyalinized by xylene, and sealed with resinene routinely.

### Immunohistochemical Staining

The expressions of LIF, CB1, and FAAH in endometrial tissue were measured by immunohistochemical staining. The immunohistochemical staining was performed using anti-CB1 (Boster, Wuhan, China), anti-LIF (Bioss, Beijing, China) and anti-FAAH antibodies (Bioworld, USA), and SP kit (ZSGB-Bio, Beijing, China) according to the manufacturer’s instruction. Paraffin sections (4 mm) were cut from the endometrial tissue, deparaffinized in xylene, rehydrated in graded alcohol, and treated with hydrogen peroxide for 10 min to block endogenous peroxidases. After antigen retrieval by target retrieval solution for 10 min, tissue sections were blocked with goat serum for 20 min. The sections were then incubated with 50 µL anti-CB1, anti-LIF, and anti-FAAH antibodies at 4°C overnight. Slides were then incubated with HRP-conjugated streptavidin (SA-HRP) at 37°C for 20 min, developed in 3,3′-diaminobenzidine chromogen (DAB, ZSGB-Bio, Beijing, China) for 6 min, counterstained with Meyer’s hematoxylin and mounted. Exclusion of the primary antibody during immunostaining was used as a negative control. The integral optical density (IOD) of the specimens were determined by CMOS-based image sensor (Olympus, Tokyo, Japan) and analyzed for the relative quantity of positive reactant.

### Statistical Analyses

All statistical analyses were calculated using SPSS13.0. The normally distributed data were presented as mean ± SD, whereas the abnormally distributed data were presented as median ± QR. Statistical significance was determined by Student’s *t*-test or Mann–Whitney *U* test. The chi-square test and the Fisher’s exact probability method were used to evaluate the data. The differences were considered to be statistically significant when *P* < 0.05.

## Results

### The General Characteristics of the Patients

The patients recruited to the study were women under the age of 38, with normal menstrual cycle and spontaneously ovulation, proposed to get IVF/ICSI treatment in our center. A total of 28 patients were included in the UI group, who had no pregnancy after IUI, excluding the uterine, endocrine, immune, and male factors. The control group included 32 patients who received IVF/ICSI treatment for the male infertility or oviduct obstruction (except for the hydrosalpinx). We first compared the basic characteristics of the patients in the two groups. As shown in Table 1, there were no statistically significant differences between two groups in the ages, durations of infertility, body mass index, and the numbers of basal follicles. In addition, the basal serum levels of follicle-stimulating hormone (7.81 ± 2.00 vs. 7.38 ± 2.24 mIU/mL), luteinizing hormone (3.67 ± 2.03 vs. 3.59 ± 2.30 mIU/mL), and estradiol (E2, 36.00 ± 19.25 vs. 42.00 ± 18.00 pg/mL) were also comparable between UI and control groups (Table 1).

**Table 1:** The basic characteristics of the participants.

	Unexplained infertility group	Control group	*P*
Age (years)	30.43 ± 2.82	30.00 ± 4.93	0.769
Duration of infertility (years)	5.36 ± 2.53	4.19 ± 3.03	0.265
BMI (kg/m^2^)	23.47 ± 3.42	23.53 ± 3.01	0.960
Number of basal follicles	9.86 ± 3.42	9.69 ± 4.09	0.904
bFSH (mIU/mL)	7.81 ± 2.00	7.38 ± 2.24	0.585
bE2 (pg/mL)^a^	36.00 ± 19.25	42.00 ± 18.00	0.560
bLH (mIU/mL)^a^	3.67 ± 2.03	3.59 ± 2.30	0.708

*^a^Used to represent non-normal data*.

*BMI, body mass index; FSH, follicle-stimulating hormone; LH, luteinizing hormone; E2, estradiol*.

### The Implantation and Pregnancy Rates Were Decreased in the UI Group

We further compared the clinical features of the two groups. As presented in Table 2, the P levels at the midluteal phase, dosages of Gn, durations of stimulation, numbers of matured follicles (diameter ≥16 mm), E2 and P levels on the day of HCG administration, the numbers of oocytes retrieved, fertilization rates and cleavage rates were not significantly different between the UI group and control group (Table 2). However, the available embryo rate (43.79%) and the embryo implantation rate (21.74%) were much lower in the UI group than those in the control group (67.38 and 56.25%). The differences of these two factors between the two groups were significant (*P* < 0.05). Moreover, the clinical pregnancy rate of the UI group (33.33%) was also significantly lower than the rate of control group (78.57%, *P* < 0.05, Table 2). These data indicated the pregnancy outcome was poor in the UI group.

**Table 2:** The clinical characteristics of the participants.

	Unexplained infertility group	Control group	*P*
P at the midluteal phase (mIU/mL)	11.51 ± 4.02	12.53 ± 4.12	0.500
Gn dosage (IU)	2,470.54 ± 799.09	2,560.94 ± 1,054.71	0.796
Duration of stimulation (days)	10.93 ± 2.02	11.38 ± 2.00	0.548
Follicle numbers on HCG day (diameter ≥16 mm)	7.57 ± 2.47	8.19 ± 3.10	0.556
E2 on HCG day (pg/mL)^a^	3,545.50 ± 2,611.25	4,953.00 ± 2,216.75	0.246
P on HCG day (ng/mL)	1.27 ± 0.25	1.15 ± 0.23	0.152
Oocytes retrieved^a^	10.50 ± 8.00	13.00 ± 9.50	0.835
Fertilization rate	84.24%	87.65%	0.363
Cleavage rate	98.71%	99.30%	1.000
Available embryo rate	43.79%	67.38%	<0.001*
Pregnancy rate	33.33%	78.57%	0.045*
Implantation rate	21.74%	56.25%	0.010*

*Statistical significance is set at P < 0.05.

*^a^Used to represent non-normal data*.

*Gn, gonadotropin; HCG, human chorionic gonadotropin; E2, estradiol; P, progesterone*.

### The Uterine Receptivity Was Reduced in the UI Group

Although patients who undergo IVF/ICSI treatment have embryos of good quality available for transfer, the embryo implantation remains the rate-limiting step in the success of therapy. One of the important factors that affect embryo implantation is the uterine receptivity, which is commonly measured by the endometrial thickness. A thin endometrium is associated with the failure of implantation and poor pregnancy outcome. We investigated whether the uterine receptivity of the UI group was decreased *via* measure of endometrial thickness. The results showed that the endometrial thickness of the UI group was lower than the thickness of control group at the midluteal phase (Table 3, UI group: 10.43 ± 1.34 mm, control group: 11.87 ± 1.36 mm), and also on the transplant day (Table 3, UI group: 11.14 ± 1.03 mm, control group: 12.00 ± 1.25 mm), suggesting the uterine receptivity was decreased in UI group compared with that of control group.

**Table 3:** The endometrial thickness.

	Unexplained infertility group	Control group	*P*
Thickness at the midluteal phase (mm)	10.43 ± 1.34	11.87 ± 1.36	0.007*
Thickness on the transplant day (mm)	11.14 ± 1.03	12.00 ± 1.25	0.042*

*Statistical significance is set at P < 0.05.

### The Levels of FAAH and LIF Were Significantly Decreased in the UI Group

**Figure 1:**
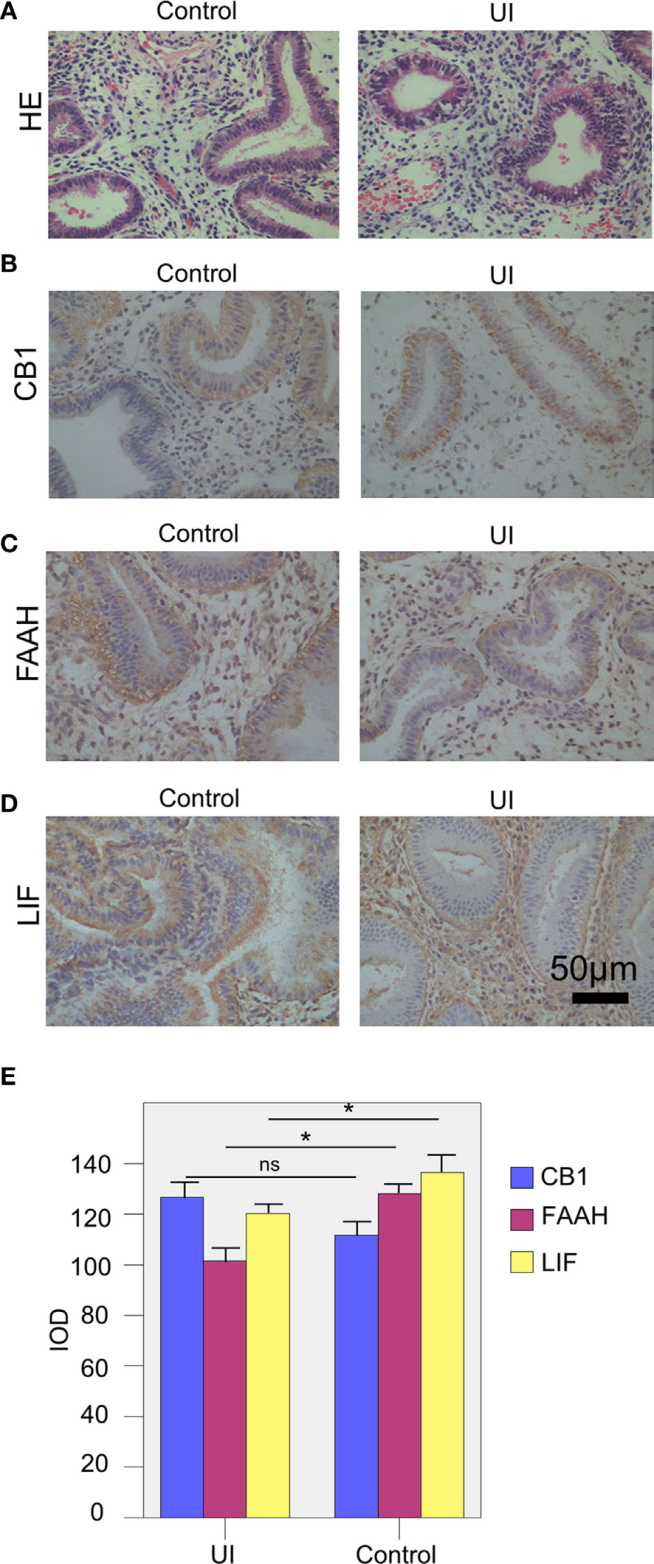
The expressions of the endocannabinoid system and leukemia inhibitory factor (LIF) of the two groups. **(A)** The HE staining of the endometrium tissue of the patients in the two groups. **(B,C)** The levels of CB1 **(B)**, fatty acid amide hydrolase (FAAH) **(C)**, and LIF **(D)** of the patients in the unexplained infertility (UI) and control groups were measured by immunohistochemical staining. **(E)** The integral optical density (IOD) values of CB1, FAAH, and LIF of UI group (*n* = 28) and control group (*n* = 32). The expressions of FAAH and LIF of UI group were significantly lower than the control group. The experiments were repeated three times. The results were presented as mean. The significance of the variation was summarized in Table 5. *P < 0.05.

A number of studies proposed a strong link between the ECS and human pregnancy; however, the physiological effects of the ECS on human reproduction are not completely understood. The ECS is composed of the endocannabinoids, cannabinoid receptors, and a variety of related enzymes, which have been found in the hypothalamus–pituitary–ovarian axis. CB1 is one of the endogenous cannabinoid receptors, widely distributed in endometrial tissue. FAAH is the material of hydrolytic enzyme endogenous cannabinoids, regulating the concentrations of endocannabinoids (22). To investigate whether there was an association between the ECS and human UI, the samples of patients in the UI and control groups were collected, and then the expressions of CB1 and FAAH in endometrial tissue were measured by immunohistochemical staining. The endometrial samples of the two groups were collected at the midluteal phase as shown by HE staining (Figure 1A). The endometrial CB1 expression (IOD) of the UI group (126.86 ± 19.03) slightly increased compared with that of the control group (111.68 ± 28.21) at the midluteal phase, but the difference between the two groups was not statistically significant (Figures 1B,E; Table 4). The endometrial FAAH expression (IOD) of the UI group (101.58 ± 28.78) was significantly lower than that of the control group (128.14 ± 24.88) at the midluteal phase (Figures 1C,E; Table 4). These data suggested there was a strong association between the FAAH expression and the UI.

**Table 4:** The integrate optical densities of CB1, FAAH, and LIF.

	Unexplained infertility group	Control group	*P*
CB1	126.86 ± 19.03	111.68 ± 28.21	0.100
FAAH	101.58 ± 28.78	128.14 ± 24.88	0.011*
LIF	120.32 ± 19.61	136.53 ± 18.97	0.029*

*Statistical significance is set at P < 0.05.

*CB1, cannabinoid type 1; FAAH, fatty acid amide hydrolase; LIF, leukemia inhibitory factor*.

The cytokines also play important roles in the human reproduction, for successful fertilization, implantation, and maintenance of early pregnancy. LIF is a member of the IL-6 family that promotes embryo implantation. We next investigated the role of LIF in human UI, and we found that the LIF expression (IOD value) of the UI group (120.32 ± 19.61) was significantly decreased compared with that of the control group (136.53 ± 18.97, Figures 1D,E; Table 4). Taken together, our data suggested that the reduction of FAAH and LIF were closely associated with the UI, which might reduce the uterine receptivity, resulted in a poor pregnancy outcome.

### AEA Has a Role on the Production of LIF by Endometrial Adenocarcinoma Cells

**Figure 2:**
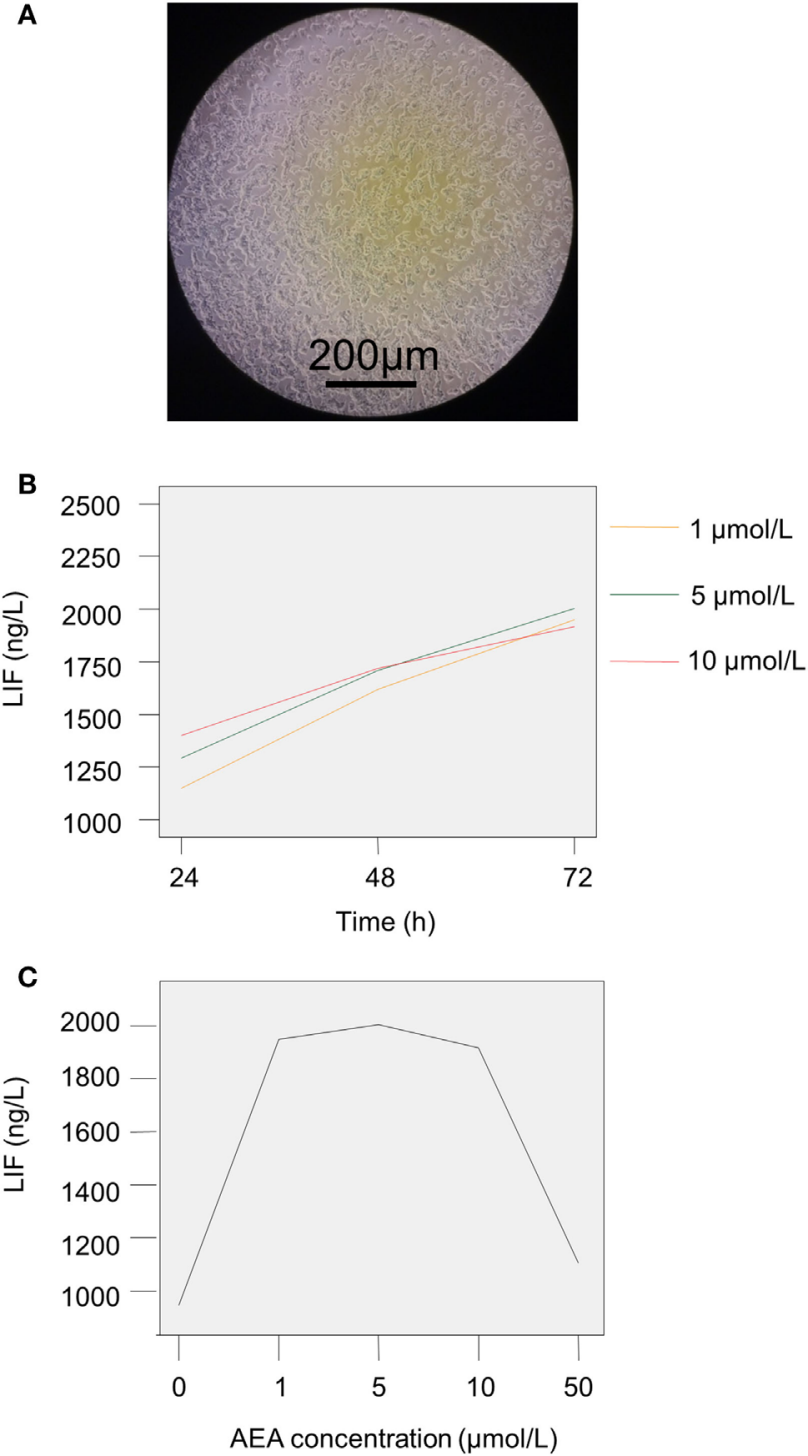
The production of leukemia inhibitory factor (LIF) by RL95-2 after the stimulation with anandamide (AEA) and cannoibind receptor agonists. **(A)** The morphology of RL95-2 in the culture. **(B)** RL95-2 was stimulated by different concentrations of AEA (1, 5, and 10 µmol/L) for 24–72 h. **(C)** The production of LIF by RL95-2 was increased when cultured with low dose of AEA (1–5 µmol/L), whereas high dose of AEA (50 µmol/L) inhibited the LIF production. The experiments were repeated three times. The results were presented as mean. The significance of the variation was summarized in Table 5. *P < 0.05.

Some studies revealed that the production of LIF by lymphoid cells could be inhibited by AEA (23). AEA is the ligand of CB1, and the level of AEA is modulated by FAAH activity (22). In the UI group, the decreased FAAH expression might attenuate the degradation of AEA, led to a reduction in LIF expression. To exam the role of AEA on the expression of LIF, we cultured human endometrial adenocarcinoma cells (RL95-2 cell line) with different concentrations of AEA, and then tested the LIF levels after 24–72 h. The RL95-2 cell line presented an adenoid structure in the culture (Figure 2A), which is a commonly used cell line to study the characteristics of endometrium due to its similar biological functions as primary endometrial epithelium cells. As shown in Figure 2B, after administration with 1–10 µmol/L of AEA, the productions of LIF by RL95-2 were increased when the incubation time was prolonged (Figure 2B; Table 5). The differences in LIF levels between each experimental group (with 1–10 µmol/L AEA stimulation for 24–72 h) and the control group (without AEA stimulation) were significant (Table 5).

**Table 5:** The LIF levels in the RL95-2 cell culture after AEA stimulation.

Group	LIF level (ng/L)
Control group	946.98 ± 27.52*
1 µmol/L AEA during 24 h	1,150.26 ± 26.16*
1 µmol/L AEA during 48 h	1,618.78 ± 33.46*
1 µmol/L AEA during 72 h	1,948.20 ± 21.81*
5 µmol/L AEA during 24 h	1,293.42 ± 15.73*
5 µmol/L AEA during 48 h	1,709.38 ± 15.98*
5 µmol/L AEA during 72 h	2,003.87 ± 37.85*
10 µmol/L AEA during 24 h	1,400.00 ± 21.36*
10 µmol/L AEA during 48 h	1,719.15 ± 56.50*
10 µmol/L AEA during 72 h	1,916.60 ± 14.05*
50 µmol/L AEA during 72 h	1,106.62 ± 21.94*

*Statistical significance is set at P < 0.05.

*There was no difference in LIF levels between either 5 µmol/L 48 h group and 10 µmol/L 48 h group, or 1 µmol/L 72 h group and 10 µmol/L 72 h group (*P* > 0.05). The differences in other groups were statistically significant*.

*AEA, anandamide; LIF, leukemia inhibitory factor*.

We further analyzed the effect of AEA concentration on the LIF level. The results showed that the LIF level was unregulated with the increased AEA level after stimulation for 24–48 h (Figure 2B; Table 5). However, after 72 h stimulation, the LIF level reached the peak (2,003.87 ± 37.85 ng/L) when the concentration of AEA was 5 µmol/L, and then the LIF level went down with the increase dosage of AEA (10–50 µmol/L, Figure 2C; Table 5). In addition, the LIF production was the lowest when stimulation by 50 µmol/L AEA for 72 h compared with other three groups (1, 5, and 10 µmol/L AEA for 72 h), and the differences were statistically significant (*P* < 0.05, Figure 2C; Table 5). These results suggested that a certain concentration of AEA might have a protective effect on implantation, while the excessive AEA might have adverse effects.

To investigate whether the modulation of LIF level by AEA was dependent on the CB1/2 receptors, we stimulated RL95-2 with AEA (5 µmol/L), AM251 (5 µmol/L), or AM630 (5 µmol/L) alone, and AEA together with AM251 or AM630 for 72 h. AM251 and AM630 are the antagonists of CB1 and CB2, respectively. The results showed that the productions of LIF by RL95-2 were significantly increased (*F* = 930.121, *P* < 0.05) with the addition of AM251 or AM630 into the culture, as well as the AEA, compared with the control group (without any stimulation, Table 6), which suggested that CB1 and CB2 might be involved in the regulation of the LIF level. Compared with the stimulation by AEA alone, the stimulations by AEA combined with AM251 or AM630 significantly reduced the LIF productions (*P* < 0.05, Table 6), which might be due to the competition effects among these molecules. These results indicated that the effects of AEA on endometrial adenocarcinoma cells were dependent on CB1 and CB2 receptors.

**Table 6:** The effects of AM251 and AM630 on LIF level.

Group	LIF level (ng/L)
Control group	935.68 ± 14.09*
5 µmol/L AEA	1,799.42 ± 19.18*
5 µmol/L AM251	1,358.04 ± 18.42*
5 µmol/L AEA and 5 µmol/L AM251	1,510.37 ± 16.77*
5 µmol/L AM630	1,353.73 ± 20.64*
5 µmol/L AEA and 5 µmol/L AM630	1,508.20 ± 21.50*

*Statistical significance is set at P < 0.05.

*There was no difference in LIF levels between either AM251 group and AM630 group, or AEA + AM251 group and AEA + AM630 group (*P* > 0.05). The differences in other groups were statistically significant*.

*AEA, anandamide; LIF, leukemia inhibitory factor*.

## Discussion

Unexplained infertility is a kind of idiopathic infertility, without pregnancy after 1 year of regularly sexual life, which cause remains unclear even though the currently advanced examination (24). UI patients usually suffer from both the financial and psychological burdens. Identification of the cause of UI could provide the theoretical basis for improving the clinical treatments, make patients to receive individualized therapy, and reduce the economic pressure of patients. Seventy-five percent of UI is caused by the failure of fertilization or implantation. The receptivity of the endometrium and the interaction between the endometrium and embryo are the major factors that affect implantation. Our study found that although the numbers of oocytes retrieved, fertilization rates and cleavage rates were comparable between the UI group and control group; the rates of available embryo, embryo implantation, and pregnancy were significantly lower than those of control group. Moreover, the endometrial thickness of the UI group was decreased at the midluteal phase, and on the transplant day, suggesting the uterine receptivity of the UI group was reduced. Taken together, our data suggested that the implantation failure resulted from poor quality of embryos or intrauterine environment might be one of the causes of UI, which provide underlying mechanisms for improving the clinical treatments to UI.

The ECS has been implicated in the mechanisms of implantation, maintenance of pregnancy, and parturition in women. AEA is considered to have an important role on the crosstalk between embryo and endometrial, which ensures the synchronous development of the preimplantation embryo and the endometrium, thereby facilitating to permit embryo implantation during the implantation window (25). FAAH is associated with the synthesis and decomposition of endocannabinoids, maintain an appropriate level of endocannabinoids suitable for embryo implantation. The dysfunction of FAAH could affect the AEA or 2-AG levels in the uterus, leading to negative effect on pregnancy outcome (26). In the menstrual cycle, the FAAH activity in peripheral blood mononuclear cells at the luteal phase (the window of implantation, day 21) was higher than the FAAH activity at other phases, whereas the AEA level was low (27). Others reported that low activity of FAAH and high AEA level were associated with the pregnancy loss after IVF-ET (IVF and embryo transfer) (28). Moreover, the FAAH activity was increased, together with a decreased AEA level, at the embryo implant site compared with those at the non-implant site (29). We reported that the FAAH expression in the endometrium of the UI group was lower than that of the control group at the mid-secretory phase, suggesting that the reduction of FAAH might lead to a decrease in the degradation of endocannabinoids, resulted in an adverse effect on the embryo implantation. CB1 was found to distribute in the brain, ovarian, endometrium, and testicle. The CB1 expression on the blastocyst was necessary for implantation; CB1 knockout mice presented a higher pregnancy loss than wild-type mice, because lack of CB1 led to the occurrence of ectopic pregnancy (30). In our study, CB1 expression of the UI group was slightly higher than that of the control group, but the difference was not statistically significant, suggesting that the implantation process was not influenced by the quantity of CB1 on the endometrial. Further investigation is required to confirm whether other receptors influence the embryo implantation.

The interaction between the ECS and immune system has many effects on the regulation of biological processes involved in implantation (31). LIF is one of the most important affecting the endometrium receptivity during the implantation window (32). It was reported that the levels of LIF and its receptor reached the peak values during the middle-late secretory phase and early pregnancy (33). Moreover, LIF knockout mice were infertile due to the implantation failure; however, LIF knockout embryos could develop in the uterus of wild-type mice (34). In addition, the frequency of relevant mutations of LIF was enhanced in infertility women, and LIF endometrial expression was impaired in UI women (20, 21, 35). We found that the endometrial LIF expression of UI patients was lower than that of control patients during the implantation window, suggesting the decreased pregnancy rate of UI patients might be due to the low receptivity of the endometrium. Taken together, the low FAAH expression in the endometrium of UI patients might lead to a high concentration of AEA; both the high AEA level and low LIF level might be harmful to the embryo implantation. The source of LIF *in vivo* was not well studied. It was reported LIF could be produced by endometrial NK cells, whether NK cells plays a major role in LIF expression and receptivity of the endometrium need to be further investigated, which might help us develop cell-specific therapies.

The results of *in vitro* culture indicated that the proper amount of AEA might help the embryo implantation, whereas a high level of AEA would have negative effects. Our results were in line with the previous study that embryo development was promoted by a low concentration of AEA, but inhibited by a high AEA level. Our data indicated that AEA might be used as a biomarker, which could help us to judge the appropriate time for embryo transfer during the IVF treatment. However, further studies are needed to confirm the appropriate level of AEA *in vivo*, which is suitable for implantation.

In summary, our study demonstrated that the expressions of FAAH and LIF were closely associated with the uterus receptivity and embryo implantation. We also established a dynamic change of LIF level with the AEA stimulation. The novel information obtained in our study should facilitate our understanding of the mechanism of ECS on human production and help with the development of ECS-based therapeutic strategies for the treatment of human infertility.

## Ethics Statement

The individual in this manuscript has given written informed consent to publish these case details. This study was approved by the ethics committee of Handan Center Hospital of Hebei Province. All procedures performed in studies involving human participants were in accordance with the ethical standards of the institutional and/or national research committee and with the 1964 Helsinki Declaration and its later amendments or comparable ethical standards.

## Author Contributions

Designed the study and wrote the manuscript: NC and GH. Performed the experiments and analyzed the data: CW, ZZ, JZ, YX, and YY. Approved the final submission: all authors.

## Conflict of Interest Statement

The authors declare that the research was conducted in the absence of any commercial or financial relationships that could be construed as a potential conflict of interest.
